# Colchicine effectiveness in symptom and inflammation modification in knee osteoarthritis (COLKOA): study protocol for a randomized controlled trial

**DOI:** 10.1186/s13063-015-0726-x

**Published:** 2015-04-30

**Authors:** Ying-Ying Leung, Julian Thumboo, Bak Siew Wong, Ben Haaland, Balram Chowbay, Bibhas Chakraborty, Mann Hong Tan, Virginia B Kraus

**Affiliations:** Duke NUS Graduate Medical School, Singapore, Singapore; Department of Rheumatology & Immunology, Singapore General Hospital, Singapore, Singapore; Department of Diagnostic Radiology, Singapore General Hospital, Singapore, Singapore; Department of Orthopaedic Surgery, Singapore General Hospital, Singapore, Singapore; The Duke Molecular Physiology Institute and Division of Rheumatology, Department of Medicine, Duke University School of Medicine, Durham, NC USA

**Keywords:** Knee osteoarthritis, colchicine, biomarkers, randomized controlled trial, double-blind, placebo-controlled, randomized

## Abstract

**Background:**

Despite the high prevalence and global impact of knee osteoarthritis (KOA), current treatments are palliative. No disease modifying anti-osteoarthritic drug (DMOAD) has been approved. We recently demonstrated significant involvement of uric acid and activation of the innate immune response in osteoarthritis (OA) pathology and progression, suggesting that traditional gout therapy may be beneficial for OA. We therefore assess colchicine, an existing commercially available agent for gout, for a new therapeutic application in KOA.

**Methods/Design:**

COLKOA is a double-blind, placebo-controlled, randomized trial comparing a 16-week treatment with standard daily dose oral colchicine to placebo for KOA. A total of 120 participants with symptomatic KOA will be recruited from a single center in Singapore. The primary end point is 30% improvement in total Western Ontario and McMaster Universities Osteoarthritis Index (WOMAC) score at week 16. Secondary end points include improvement in pain, physical function, and quality of life and change in serum, urine and synovial fluid biomarkers of cartilage metabolism and inflammation. A magnetic resonance imaging (MRI) substudy will be conducted in 20 participants to evaluate change in synovitis. Logistic regression will be used to compare changes between groups in an intention-to-treat analysis.

**Discussion:**

The COLKOA trial is designed to evaluate whether commercially available colchicine is effective for improving signs and symptoms of KOA, and reducing synovial fluid, serum and urine inflammatory and biochemical joint degradation biomarkers. These biomarkers should provide insights into the underlying mechanism of therapeutic response. This trial will potentially provide data to support a new treatment option for KOA.

**Trial registration:**

The trial has been registered at clinicaltrials.gov as NCT02176460. Date of registration: 26 June 2014.

**Electronic supplementary material:**

The online version of this article (doi:10.1186/s13063-015-0726-x) contains supplementary material, which is available to authorized users.

## Background

Knee osteoarthritis (KOA) is one of the five leading causes of disability among noninstitutionalized adults [1]. The risk of mobility disability (defined as needing help walking or climbing stairs) attributable to OA alone is greater than any other medical condition in people aged 65 and over [2]. In the World Health Organization Global Burden of Disease Study of 21 epidemiological regions across the world, from 1990 to 2010 there was a 26.8% increase in the burden of KOA as measured by years lived with disability per 100,000 persons [3]. The global prevalence of KOA will continue to rise in tandem with increases in the prevalence of obesity and longevity worldwide. Despite the high prevalence and global impact of KOA, current treatments are limited to palliative measures broadly focused on analgesia, and when this fails, surgical knee replacement. Given its frequency, associated disability and societal cost, patients with KOA are in urgent need of effective treatment that reduces pain and symptoms, and slows progression of OA. There are a number of agents, like glucosamine sulfate, chondroitin sulphate, diacerin, doxycycline and licofelone that have demonstrated structural modifying capabilities in clinical trials [4]. Overall, these trials did not report a favorable profile with respect to side effects, no concomitant symptomatic relief or failed to demonstrate the structural modifying endpoints as required by the Food and Drug Administration (FDA) [5].

In a KOA cohort without clinical evidence or self-report of gout, we found a significant correlation of synovial fluid uric acid with radiographic and scintigraphic measures of OA severity [6]. We also observed strong correlations of radiological OA severity and synovial fluid uric acid with synovial fluid interleukin (IL)-18 and IL-1β; these two cytokines are classically produced during gout attacks by innate immune system activation mediated by uric acid crystal-induced inflammasome assembly in macrophages. These results strongly support the involvement of uric acid and the innate immune system in OA pathology and progression. The pain and symptom relieving effects of colchicine for KOA have been demonstrated in three small, but well-performed human randomized controlled trials (RCTs) [7-9]; however, the mechanism of action of colchicine in KOA has never been evaluated. We hypothesize that colchicine will block inflammasome-mediated inflammation, thereby improve the signs and symptoms of OA, and reduce synovial fluid serum and urine inflammatory and biochemical joint degradation biomarkers. We propose a randomized controlled trial (RCT) of colchicine, an agent traditionally used for the treatment of gout, to examine the effects on signs and symptoms of KOA and to evaluate the mechanism of action of this therapy in OA through analysis of synovial fluid and systemic biomarker profiles. The protocol of the COLKOA trial is outlined in this paper.

## Methods/Design

### Trial objectives

The trial is designed to address the following objectives:

1. To determine whether daily oral colchicine at standard clinical doses (0.5 mg two times daily), compared to placebo, can decrease the pain of symptomatic KOA and improve function when used in addition to the patient’s current stable analgesic regimen.
2. To evaluate the mechanism of action of colchicine for reducing KOA signs and symptoms through analyses of synovial fluid, serum, and urine biomarker profiles; characterizing the state of joint tissue metabolism (through joint degradation and synthesis markers), inflammatory mediators of the innate immune system and the NACHT-LRR-PYD-containing protein-3 (NALP3) inflammasome before and after colchicine treatment compared to placebo.

### Study design

COLKOA is a single center, double-blind randomized placebo-controlled clinical trial, conducted at Singapore General Hospital, comparing colchicine with placebo in patients with primary KOA. The study protocol was approved by the SingHealth Centralized Institutional Review Board (ref. CIRB2012/659/E), and informed consent will be obtained from all participants before the start of the study. A clinical trial certificate (CTC1300061) was obtained from the Singapore Health Sciences Authority.

### Participant selection and eligibility criteria

**Figure 1:**
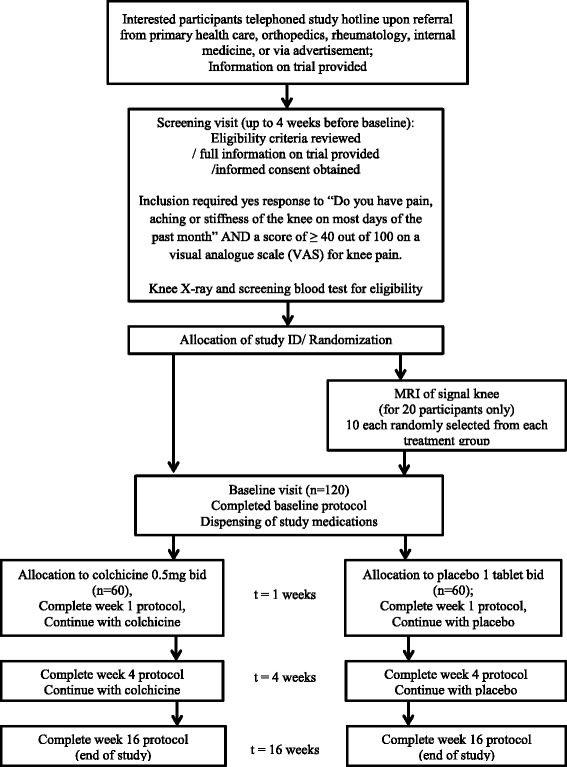
Flow chart of the study. ID, identification; MRI, magnetic resonance imaging; bid, twice per day.

Adults aged 21 to 79 with symptomatic KOA will be recruited through advertisements, referrals from primary health care, orthopedic and internal medicine specialists. Study inclusion requires the following: symptomatic KOA meeting American College of Rheumatology (ACR) criteria [10]; radiographic criteria for KOA with Kellgren-Lawrence (KL) stage of ≥2 in at least one knee at screening [11]; symptomatic KOA defined as a positive response to the question, “Do you have pain, aching or stiffness of the knee on most days of the past month; and a score of ≥40 out of 100 on a visual analog scale (VAS) for knee pain. Further details regarding inclusion and exclusion criteria are summarized in Table 1. We also excluded subjects on current treatment with drugs known to inhibit cytochrome 450 (CYP3A4) and/or P-glycoprotein (P-gp) (see Additional file 1: Table S1) [12] that increase the risk of colchicine-induced toxic effects. Current treatment with these drugs mandates exclusion from the study until the drug is discontinued >14 days and no future treatment anticipated. As grapefruit is a moderate CYP3A4 inhibitor, and colchicine toxicity has been reported in a patient consuming it concomitantly, participants in this study have to agree to avoid consuming grapefruit and grapefruit juice during the course of the RCT. The study design and schedule are summarized in Figure 1 and Table 2.

**Table 1:** **Inclusion and exclusion criteria for colchicine effectiveness in symptom and inflammation modification in knee osteoarthritis (COLKOA)**

Inclusion Criteria	• Symptomatic KOA meeting American College of Rheumatology (ACR) criteria
	• Radiographic criteria for KOA with Kellgren-Lawrence (KL) stage of ≥ 2 in at least one knee
	• Response positive to the question "do you have pain, aching or stiffness of the knee on most days of the past month
	• Score of ≥ 40 out of 100 on a visual analogue scale (VAS) for knee pain
	• Age 21 years or above
	• Male and female subjects and all ethnicities included
	• Patients to agree to avoid consuming grapefruit and grapefruit juice while using colchicine
	• Ability to provide informed consent
Exclusion Criteria	• Exposure to a corticosteroid (either parenteral or oral) within 3 months prior to the study enrolment
	• Knee arthroscopic surgery within 6 months prior to the study enrolment
	• Known history of avascular necrosis, inflammatory arthritis (e.g. Rheumatoid Arthritis), Paget's disease, joint infection, periarticular fracture, neuropathic arthropathy, reactive arthritis, or gout involving the knee
	• Contraindication to arthrocentesis (warfarin use, bleeding disorder, skin rash or skin infection of signal knee)
	• Knee joint replacement in either knee
	• History of podagra, active gout or treatment for gout
	• Pregnancy or lactation - women of childbearing potential will have serum pregnancy testing (ßHCG) at time of entry prior to any imaging studies (radiographic or MRI); female subjects of childbearing potential must agree to use some form of contraception during the 16 week trial and for 1 week after the end of the trial (over 6 half-life equivalents)
	• Renal failure with serum creatinine > 150 mmol/L (1.7 mg/dL);
	• Hepatic impairment defined by serum alanine transaminase (ALT) above the upper limit of normal (ULN) for the clinical laboratory performing the screening test
	• Muscle impairment defined by elevated serum creatine phosphokinase (CPK) above the ULN for the clinical laboratory performing the screening test
	• Personnel directly affiliated with this study or their immediate family members (defined as a spouse, parent, child or sibling, whether biological or legally adopted)
	• Current enrollment in or discontinued within the last 30 days from a clinical trial involving an off-label use of an investigational drug or device, or are concurrently enrolled in any other type of medical research judged to be scientifically or medically incompatible with this study
	• Inability to understand and cooperate with the investigators or to give valid consent;
	• Contraindication for magnetic resonance imaging (MRI) - this is exclusion only for the subset of individuals selected for this imaging procedure
	• Anticipation of need for joint replacement within 4 months of the start of the intervention
	• Current treatment with drugs known to inhibit CYP3A4 isoforms and/or P-glycoprotein (P-gp) that increase the risk of colchicine-induced toxic effects

**Table 2:** **Schedule of assessment and data collection**

	**Screening visit**	**Allocation**	**Post allocation**			
**Time point**	**Up to 4 weeks before BL**	**0**	**Baseline visit (BL)**	**Week 1**	**Week 4**	**Week 16 (end)**
**Enrolment:**						
Eligibility screening	x					
Informed consent	x					
Screening knee X-ray	x					
Screening blood test *	x					
Physical Examination	x		x	x	x	x
**Interventions:**						
Colchicine 0.5 mg bid			Start here			End here
Placebo 1 tablet bid			Start here			End here
**Assessments:**						
Physical Examination	x		x	x	x	x
Full blood count	x					x
Renal function test	x			x	x	x
Liver function test	x			x	x	x
Uric acid	x			x	x	x
CPK	x			x	x	x
βHCG (for women with child bearing potential)	x					
Blood biomarkers collection			x	x	x	x
Urine biomarkers collection			x	x	x	x
Blood for pharmcogenomic			x			
Colchicine level			x	x	x	x
Both knees aspiration			x			x
Knee X-ray (both sides)	x					
MRI of signal knee ǂ (for 20 subjects only)		x				
Adverse event enquiries			x	x	x	x
Enquiries to compliance to usual medications for knee			x	x	x	x
Clinical data collection			x			x
Pain score enquiries (signal knee)			x	x	x	x
Demographic data collection			x			
WOMAC			x			x
HAQ			x			x
SF36v2			x			x

*(FBC, renal function, liver function, uric acid, CPK, βHCG for women with child bearing potential); ǂ: For ten colchicine treated and ten placebo treated participates, randomly assigned; CPK, creatine phosphokinase; βHCG, β-human chorionic gonadotropin; MRI, magnetic resonance imaging; X-ray, radiography; WOMAC, Western Ontario and McMaster Universities Osteoarthritis Index; HAQ, Health Assessment Questionnaire; SF36v2, Medical Outcome Short Form 36 version 2.

### Treatment assignment and allocation concealment

The US Food and Drug Administration (FDA) and Singapore Health Sciences Authority approved colchicine for the treatment of acute flare-ups of gout and for chronic treatment of Familial Mediterranean Fever (FMF). Colchicine is available as a generic medication in Singapore. Generic colchicine (SIN 12490P) are repackaged for use in this study. Identical placebo tablets are produced to ensure allocation concealment. Upon production, study medications are packaged and relabeled into numbered bottles by designated pharmacists according to the randomization list prepared by the trial biostatistician. Participants are randomly assigned in blocks of ten to the intervention arm or the placebo arm in a 1:1 ratio.

### Blinding

Investigators and participants will remain blinded throughout the trial. Emergency unblinding will be allowed in limited situations that are perceived to impact the safety of study participants. Code-break envelopes for the full randomization schedule will be maintained by the designated biostatistician.

### Intervention

Participants with symptomatic KOA will be randomized to oral colchicine 0.5 mg or placebo twice daily for 16 weeks. Participants are permitted to remain on their baseline adjunctive therapy, including topical analgesics, supplements, nonsteroidal anti-inflammatory drugs (NSAIDs) without changes of drug and dosage for the duration of the study. They will also be allowed the use of paracetamol <2 g/day as rescue analgesia. Pill counting at the end of the study will be conducted to determine the amount of study drug and rescue medicine utilized over the course of the 16-week study. Participants are asked not to start any new pharmacological and nonpharmacological therapies for their KOA, including new topical medications, acupuncture, exercise class or physiotherapy, for the duration of the study. Detailed information on existing or new medication use for knee symptoms is collected at weeks 1, 4 and 16.

### Clinical variables

Clinical variables include age, sex, ethnic group, educational level, history of major joint injuries, history of knee joint surgery, comorbidities, and current medications and dosage, presence or absence of pain in other joints; severity of pain of the signal knee and non-signal knee at rest and on movement. Physical examination will include measurement of joint alignment [13] and range of motion of the signal knee. The signal knee is defined as the one meeting entry criteria; if both knees meet entry criteria, the more symptomatic knee at baseline will be considered the signal knee; if symptoms are equal on both sides, the dominant knee will be designated the signal knee. Patient reported outcome measures include Western Ontario and McMaster Universities Osteoarthritis Index (WOMAC) [14], Health Assessment Questionnaire (HAQ) [15] and Outcome survey short-form 36 version 2 (SF36v2) [16]. All clinical variables are obtained at baseline and week 16, the end of the study.

### Biospecimen collection

Prior to randomization, participants will have simultaneous blood and urine collection and joint fluid aspiration from both knees by an experienced rheumatologist. Blood samples are obtained at least 2 hours post-prandial, and urine samples are obtained from the second void of the morning. The blood and urine sample collection is repeated at week 1, week 4 and the end of study (week 16). Synovial fluid collection is repeated at the end of study. Synovial fluid is aspirated directly from each knee by a 20-gauge needle. If no fluid is obtained, 10 ml of sterile saline is injected into the joint and the fluid aspirated after gentle compression of the knee. The dilution factor of the lavage fluid (that obtained by saline injection) is determined according to an established urea method [17].

### Soluble biomarker variables

In addition to uric acid, four types of soluble biomarkers will be assessed: inflammasome associated biomarkers, biomarkers of oxidant stress, other inflammatory biomarkers, and cartilage degradation biomarkers. The serum (s) and synovial fluid (sf) markers to be measured by category are the following: s/sf uric acid; inflammasome associated (s/sf IL-1 ß , IL-18, tumor necrosis factor (TNF)α, caspase-1, Toll-like receptor (TLR)-2, and TLR-4); oxidative stress (s/sf xanthine oxidase, allantoin, inorganic pyrophosphate (PPi) and sf carbonyl content); other inflammatory markers (sCD163 which potentially indicative of macrophage activation, s/sf matrix metalloproteinase (MMP)3, IL-6, and high sensitivity C-reactive protein (hs-CRP)); cartilage degradation markers (sf glycosaminoglycan fragments and sf C-terminal crosslinked telopeptide type II collagen (CTXII)). In addition, s/sf urea will be measured to correct sf biomarker concentrations for any dilution introduced by lavage.

### Magnetic resonance imaging variables

The specific subjects selected for MRI were pre-specified, without knowledge of their clinical data, as the 6th to 25th participants being randomized to colchicine and placebo respectively. This choice ensured, based on block randomization, that half (n = 10) would be colchicine-treated and half (n = 10) placebo-treated. The MRI examinations consist of a standardized set of acquisitions with specific positioning and localization requirements. The MRI imaging will be performed using a 3 Tesla Siemens Skyra (2012, Simens, Erlangen, Munich, Germany) with the signal knee immobilized in a 15 Channel Transmit/ Receive Knee coil. The intravenous contrast medium used will be Dotarem, 0.5 mmol/ml (Gadoteric Acid, Guerbet, France) with a maximum administered volume of 10 ml. The whole knee joint will be scored by an experienced musculoskeletal radiologist blinded to clinical details of participants, using the Boston Leeds Osteoarthritis Knee Score (BLOKS) [18]. At the time of the screening evaluation, knee radiography of all patients will be performed with the SynaFlexor platform and variable beam angle as previously described [19] to optimize images for serial joint space width measurements in the event of any future long-term follow-up of the participants in this trial.

### Safety evaluation

Safety and tolerability of treatment will be assessed at weeks 1, 4 and 16. Safety is assessed by identifying adverse events (AEs) during treatment using open-ended questions and a checklist that includes diarrhea, myalgia and muscle weakness, and physical examination of muscle tenderness and muscle strength. Laboratory assessments include renal and liver function tests and creatine phosphokinase (CPK). All patients are given a 24-hour emergency contact number for enquiries should there be adverse events. Patients who are noted to have significant adverse events at week 4, such as myalgia or an increase in arthralgia will be contacted by the study team via telephone and arrangements will be made for extra outpatient visits as clinically indicated. Adverse events are recorded at each visit and up to 16 weeks and are analyzed for their seriousness, intensity and causal relationship with treatment and outcome according to the National Institutes of Health standardized Common Terminology Criteria [20]. A Data Safety and Monitoring Board consisting of two independent rheumatologists and one biostatistician will monitor the study biannually and review reportable adverse events and be responsible for the decision to continue the study in the event of a drug related serious adverse effect.

### Outcomes

The primary endpoint will be 30% improvement in total WOMAC score of the signal knee. Secondary endpoints include change in WOMAC pain score and physical function score, HAQ, SF36v2 and the quantity of rescue medication used. Other secondary endpoints include treatment response defined by the Outcome Measures in Rheumatology Clinical Trials and Osteoarthritis Research Society International (OMERACT-OARSI) 2004 criteria [21], wherein participants are classified as responders if the pain or physical function score is decreased by ≥30% and by at least 20 mm on the VAS. Other endpoints include statistically significant (*P* <0.05) treatment related change in MRI detected synovitis and cartilage morphology and decline in synovial fluid IL-18, IL-1β, or TNF-α. We will also explore the polymorphisms of multidrug resistance gene-1 (MDR-1) genes (1236C > T, 2677G > A/T, 3435C > T) that encodes P-gp and cytochrome CYP3A4*1B, CYP3A5*3 and CYP3A5*6; corresponding to treatment response to colchicine and drug levels in serum and synovial fluid.

### Sample size estimation

Based on the results of an existing RCT [8], we expect to achieve a 30% improvement rate in WOMAC in 57% and 23% of the subjects in the colchicine and placebo groups, respectively. To detect this difference at a significance level of 5% (*P* <0.05) and 80% power, 32 participants per group (64 total) will be required; for 90%, 42 participants per group (84 total) will be required. We therefore plan to enroll 120 participants for this trial to insure 90% power even with a dropout rate of up to 30%.

### Statistical analysis

Analyses will be conducted using R 3.1.1 (R Core Team, Vienna, Austria). Throughout, *P* values <0.05 will be considered statistically significant. Analyses will be performed from an intention-to-treat perspective. The proportion of participants achieving the primary endpoint, 30% improvement in total WOMAC at week 16, will be compared between the colchicine and placebo groups using a chi-squared test. Participants who do not have a follow-up WOMAC will be assumed to not have a ≥30% improvement in WOMAC, thereby making the analysis conservative. Additionally, WOMAC score, as well as other secondary endpoints, for each patient across time will be analyzed in the context of generalized estimating equations (GEE) models with sandwich estimators of variance to appropriately handle the dependency among multiple observations on the same patient. While randomization ensures approximate balance with respect to known and unknown confounders, this GEE analysis will be adjusted by potential confounders including age, gender, and baseline clinical covariates. These adjustment confounders will be selected by stepwise variable selection with the Akaike information criterion (AIC). While attrition is not expected to be high, the GEE model is able to utilize all nonmissing data while remaining unbiased so long as the pattern of data missingness, conditional on observed outcomes and covariates, does not depend on the values of the missing data, that is, the data must be missing at random (MAR). If it is discovered that the pattern of data missingness depends on covariates not included in the adjusted model, these variables may also be incorporated so that the MAR assumption is satisfied [22]. Variables associated with the pattern of data missingness would be incorporated using a forward stepwise approach with a *P* value <0.05 entry criterion. Secondary endpoints will be analyzed in the context of GEE models, depending on whether the endpoint is categorical or continuous, with a fixed effect for treatment. Secondary endpoints that are measured on both of a patient’s knees will additionally include fixed effects for KOA and KOA by treatment interaction. Secondary analyses will also be supplemented by covariate adjusted tests, where covariates will once again be selected using stepwise variable selection with AIC. To evaluate the mechanism of action of colchicine for reducing knee OA signs and symptoms, the change from baseline to 16 weeks (at study end) in synovial fluid, serum, and urine biomarker profiles will be compared between oral colchicine and placebo treatment. The relationship between response (≥30% improvement in WOMAC) and change from baseline to 16 weeks (at study end) in synovial fluid, serum, and urine biomarker profiles will be assessed in the context of a logistic regression model. Various exploratory statistical models and analyses such as structural equation modelling and factor analysis will be performed to further elucidate the mechanisms of action of colchicine.

## Discussion

This RCT is proposed at a time when effective treatments for OA are urgently needed. It is well grounded in a strong biological rationale and a strong clinical rationale with the several small preceding RCTs of colchicine for KOA. This proposed RCT is two- to threefold larger than any of the existing trials allowing greater power to test and validate the clinical effectiveness of colchicine for knee OA signs and symptoms. This RCT will break new ground by exploring the mechanism of action of colchicine in knee OA through testing the ability of colchicine to inhibit inflammasome activation in OA. Finally and importantly, this trial can serve as a valuable paradigm for a subsequent larger longer-term multicentered RCT with long-term radiographic and MRI follow-up to evaluate the effects of colchicine to slow or halt progression of OA structural deterioration in addition to evaluating its long-term effects on OA symptoms and function.

**Figure 2:**
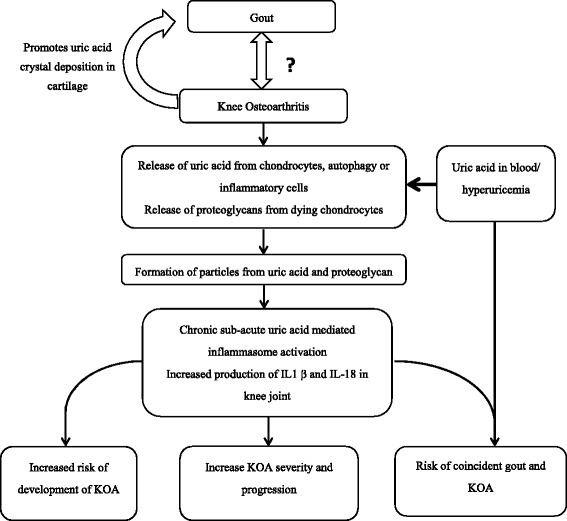
Schematic diagram showing interaction of osteoarthritis (OA) and uric acid. Adapted from Denoble *et al*. [6] Figure legends: In this model, osteoarthritis (OA) leads to release of uric acid and proteoglycans, which enucleate to form microparticles. Hyperuricemia further increases diffusion of uric acid into joint fluid from blood, contributing to constitutive sub-acute inflammation and progression of OA via inflammasome activation. IL, Interleukin; KOA, knee osteoarthritis.

The association of uric acid and OA has long been observed [23], although not statistically significant after controlling for body mass index in cohort studies [24-26]. One study noted that acute attacks of gout coincided with the presence of clinical OA in the same joint suggesting that OA may facilitate the localized deposition of monosodium urate (MSU) crystals [27]. In a recent study of cadaveric ankles (talus surface only), crystals were strongly associated with cartilage lesions - overall 92% of cartilage lesion cases were associated with crystal deposition of which 67% and 33% were MSU and calcium pyrophosphate dihydrate (CPPD) crystals, respectively [28]. Apart from the morphological changes, crystal deposition in joints was also associated with immunohistochemical changes including superficial zone protein and collagen X that is associated with cartilage degeneration and repair. A study of human chondrocyte explants also demonstrated that MSU crystals inhibit chondrocyte viability and function [29]. In a study among OA subjects without clinical gout, we recently demonstrated a strong positive association between synovial fluid uric level, IL18 and IL1β and OA severity as measured by imaging [6]. This suggested that MSU crystals may contribute both to the initiation and propagation of cartilage degradation. We hypothesize in OA that the degradation of cartilage extracellular matrix components may facilitate the nucleation of MSU crystals, forming microparticles that trigger the innate immune response via activation of dendritic cells and macrophages with subsequent priming of T cells (Figure 2).

This new conception of the pathogenesis of OA has very important treatment implications; it suggests that existing therapies for gout may be of benefit in OA. One such long-standing therapy for gout, colchicine, effectively suppresses the innate immune response at various levels; this makes colchicine a strong candidate as an effective treatment. At micromolar concentrations (from 0.5 μM to 5 μM) colchicine has several anti-inflammatory mechanisms of action: it suppresses activation of the MSU crystal-induced NALP3 inflammasome, and it suppresses IL-1β processing and release and neutrophil recruitment. It is important to realize that colchicine preferentially accumulates in leukocytes. The concentration in neutrophils may be more than 16 times the peak concentration in plasma. It is possible that a continuous low prophylactic dose of colchicine can achieve an intracellular concentration in macrophages high enough to inhibit NALP3 inflammasome activation that is triggered by danger signals formed after release of uric acid and cartilage extracellular matrix fragments from degrading cartilage in OA.

At much lower (nanomolar) concentrations, colchicine has additional mechanisms of action: it blocks the release of a crystal-derived chemotactic factor from neutrophil lysosomes; and it blocks neutrophil adhesion to endothelium. In an *in vivo* study colchicine inhibits MSU crystal-induced production of superoxide anions from mouse neutrophils and macrophages at doses 100 times lower (given by oral gavage at doses of 0.05 μmol/kg, 4 hours prior to stimulation) than those required for inhibition of neutrophil infiltration (oral gavage at 5 μmol/kg) [30]. This suggests that colchicine at the doses administered in this RCT may counter inflammation through antioxidant effects related to inhibition of superoxide anion. The effectiveness of colchicine in KOA has been demonstrated in preceding small RCTs. One of these RCT was done in subjects with signs of knee inflammation, while the other two were among subjects with primary KOA [7-9]. The main limitations in these RCTs include single-centered studies with small sample sizes. One study was in a subgroup of patients who had significant knee effusions who received intra-articular corticosteroids, thus limiting its generalizability [7]. Two studies did not use standardized outcome measures [7,9], and in all studies, the underlying mechanism of colchicine on KOA were not evaluated. The long-term safety profile of colchicine is well known based on its traditional use as a treatment for gout, its evaluation in clinical trials used for marketing approval of colchicine for gout [31], and its use as the standard first line and long-term treatment for FMF [32-34]. An additional recent RCT, evaluating colchicine at 0.5 mg/day for 3 years in addition to statins and standard therapies, appeared effective for the prevention of cardiovascular events in participants with stable coronary disease and also demonstrated an excellent safety profile [35]. Apart from the US, another distinct advantage of colchicine is its availability as generic medication. If this proof of concept study is successful, it may potentially benefit a large OA patient population at an affordable price.

There are a few limitations of this study. The development of disease-modifying osteoarthritis drugs (DMOADs) is faced with many challenges [4]. For instance, the current FDA guidelines define the current acceptable structural endpoint for DMOAD clinical trials as a slowing in the loss of knee joint space narrowing (JSN) using radiographs, which in addition, would need to be accompanied by symptom improvement [36]. Although we may break new ground in understanding the underlying mechanism of colchicine and clinical response to colchicine treatment in the COLKOA trial, there is no structural endpoint in this study appropriate for meeting the guideline criteria for DMOAD. Nevertheless, this study will provide valuable information regarding the justification for a subsequent larger multi-center DMOAD trial with structural endpoints and insights into study design. Other limitations include the use of frequent and moderate knee pain as a recruitment criterion, which may inflate response rates in both treatment and placebo groups due to regression to the mean. However, patients with higher pain scores constitute the population of interest and randomization ensures that there will be no systematic difference between the arms other than treatment versus placebo.

In summary, KOA is disabling and is increasingly prevalent. A DMOAD treatment to relieve symptoms and progression is urgently needed. Colchicine has theoretical and some clinical evidence of benefit in subjects with KOA. It has a good safety profile and is practical for long term use. The COLKOA trial will stringently test its clinical efficacy and underlying mechanism of action. This will potentially provide a new and affordable treatment for KOA.

### Trial status

ClinicalTrials.gov Identifier: NCT02176460. Recruitment for this trial started in October 2013. As of December 2014, 83 participants have been recruited and 61 participants have completed the trial protocol. At the time of manuscript submission, recruitment and follow-up of patients is in progress.

## Additional file

Additional file 1: Table S1. Drugs that interact negatively with colchicine [12].

## Abbreviations

ACR: American College of Rheumatology
AEs: adverse events
AIC: Akaike information criterion
ALT: alanine transaminase
CPK: creatine phosphokinase
CPPD: calcium pyrophosphate dehydrate
Cmax: mean peak concentration
CTXII: C-terminal crosslinked telopeptide type II collagen
CYP3A4: Cytochrome 450 3A4
DMOAD: disease modifying anti-osteoarthritis drug
FDA: Food and Drug Administration
FMF: familial Mediterranean Fever
HAQ: Health Assessment Questionnaire
Hs-CRP: high sensitivity C-reactive protein
IL: interleukin
KL: Kellgren-Lawrence staging of radiographic OA
KOA: knee osteoarthritis
MAR: missing at random
MDR-1: multidrug resistance gene-1
MMP: matrix metalloproteinase
MRI: magnetic resonance imaging
MSU: monosodium urate
NALP3: NACHT-LRR-PYD-containing protein-3 NSAIDs, nonsteroidal anti-inflammatory drugs
OMERACT- OARSI: Outcome Measures in Rheumatology Clinical Trials and Osteoarthritis Research Society International criteria
P-gp: P-glycoprotein
PPi: inorganic pyrophosphate
RCT: randomized controlled trial
SF36v2: Outcome Survey Short-form 36 version 2
S: Serum
Sf: synovial fluid
TLR: toll-like receptor
TNF: tumor necrosis factor
ULN: upper limit of normal
VAS: visual analog scale
WOMAC: Western Ontario and McMaster Universities Osteoarthritis Index
