# Supplementary material for: Colchicine effectiveness in symptom and inflammation modification in knee osteoarthritis (COLKOA): study protocol for a randomized controlled trial
Source: Trials. 2015 Apr 30;16:200. doi: 10.1186/s13063-015-0726-x (PMC4434529; doi:10.1186/s13063-015-0726-x)
Supplement: Additional file 1: Table S1. — Drugs that interact negatively with colchicine [12]. [file 13063_2015_726_MOESM1_ESM.docx]

Supplementary Table 1. Drugs that interact negatively with colchicine [12]

| Drug | Noted or anticipated outcome |
| --- | --- |
| **Strong CYP3A4 Inhibitors** |  |
| atazanavir, clarithromycin, indinavir,  itraconazole, ketoconazole, nefazodone,  nelfinavir, ritonavir, saquinavir, telithromycin | Significant increase in colchicine plasma levels; fatal colchicine toxicity has been reported with clarithromycin, a strong CYP3A4 inhibitor. Similarly, significant increase in colchicine plasma levels is anticipated with other strong CYP3A4 inhibitors. |
| **Moderate CYP3A4 Inhibitors** |  |
| amprenavir, aprepitant, diltiazem,  erythromycin, fluconazole, fosamprenavir,  grapefruit juice, verapamil | Significant increase in colchicine plasma concentration is anticipated. Neuromuscular toxicity has been reported with diltiazem and verapamil interactions. |
| **P-gp Inhibitors** |  |
| cyclosporine, ranolazine | Significant increase in colchicine plasma levels; fatal colchicine toxicity has been reported with cyclosporine, a P-gp inhibitor.  Similarly, significant increase in colchicine plasma levels is anticipated with other P-gp inhibitors. |

Current treatment with these drugs mandate exclusion from the study until the drug is discontinued >14 days and no future treatment anticipated.
